# Development of Gentle Slope Light Guide Structure in a 3.4 μm Pixel Pitch Global Shutter CMOS Image Sensor with Multiple Accumulation Shutter Technology †

**DOI:** 10.3390/s17122860

**Published:** 2017-12-09

**Authors:** Hiroshi Sekine, Masahiro Kobayashi, Yusuke Onuki, Kazunari Kawabata, Toshiki Tsuboi, Yasushi Matsuno, Hidekazu Takahashi, Shunsuke Inoue, Takeshi Ichikawa

**Affiliations:** Canon Inc., 70-1, Yanagi-cho, Saiwai-ku, Kawasaki-shi, Kanagawa 212-8602, Japan; kobayashi.masahiro@canon.co.jp (M.K.); onuki.yusuke@canon.co.jp (Y.O.); kkawabata@cusa.canon.com (K.K.); tsuboi.toshiki034@canon.co.jp (T.T.); matsuno.yasushi@canon.co.jp (Y.M.); takahashi.hidekazu@canon.co.jp (H.T.); inoue.shunsuke@canon.co.jp (S.I.); ichikawa.takeshi@canon.co.jp (T.I.)

**Keywords:** Global Shutter, Light Guide, Sensitivity, Parasitic Light Sensitivity

## Abstract

CMOS image sensors (CISs) with global shutter (GS) function are strongly required in order to avoid image degradation. However, CISs with GS function have generally been inferior to the rolling shutter (RS) CIS in performance, because they have more components. This problem is remarkable in small pixel pitch. The newly developed 3.4 µm pitch GS CIS solves this problem by using multiple accumulation shutter technology and the gentle slope light guide structure. As a result, the developed GS pixel achieves 1.8 e^−^ temporal noise and 16,200 e^−^ full well capacity with charge domain memory in 120 fps operation. The sensitivity and parasitic light sensitivity are 28,000 e^−^/lx·s and −89 dB, respectively. Moreover, the incident light angle dependence of sensitivity and parasitic light sensitivity are improved by the gentle slope light guide structure.

## 1. Introduction

**Figure 1:**
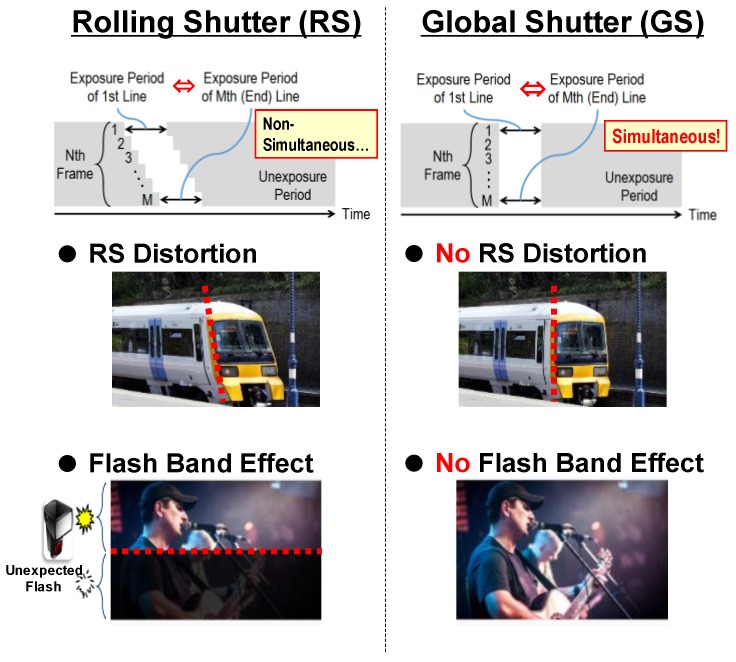
Rolling shutter problems.

**Figure 2:**
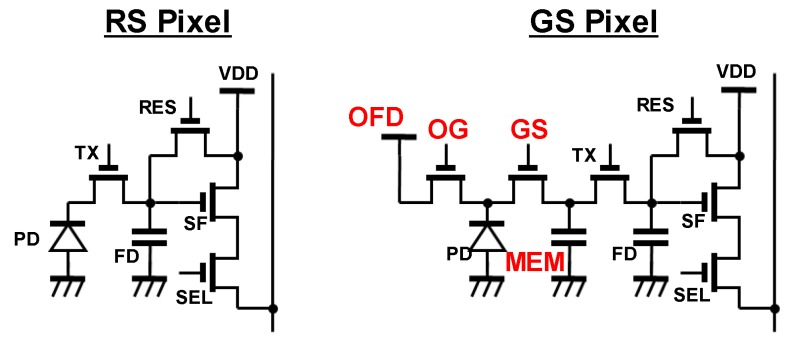
Pixel schematic diagram of RS pixel and GS pixel.

CMOS image sensors (CISs) with global shutter (GS) function are strongly required for use in broadcasting, automobile, drones and surveillance applications, in order to avoid image degradation caused by rolling shutter (RS) distortion (Figure 1). On the other hand, high image quality is demanded in CISs for those applications. To realize GS CISs, a memory structure (MEM), additional MOS transistors (Overflow gate (OG), GS) and additional drivelines (OG, GS, Overflow drain (OFD)) are necessary in each pixel (Figure 2). Due to this increase in the number of components, photodiode (PD) area and aperture size are restricted. Therefore, sensor performance (e.g., noise, sensitivity and saturation) of GS CISs has generally remained inferior to that of RS sensors. To break down this problem, the GS sensors implementing various techniques have been recently proposed [1,2,3,4]. We have also developed GS sensors that adopt the multiple accumulation shutter technology [5] and light guide structure [6,7]. These sensors have been reported to have superior characteristics. In this paper, we describe further details of a multiple-accumulation shutter technology and a gentle slope light guide (LG) structure for small GS pixel and introduce the developed 3.4 μm pitch global shutter with these techniques. 

## 2. Sensor Architecture

**Figure 3:**
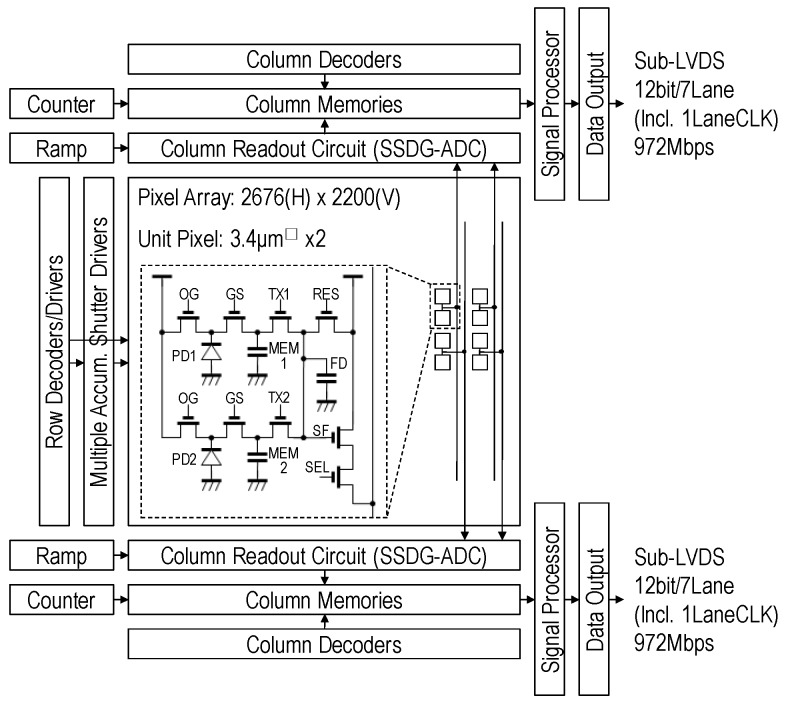
GS CIS block diagram.

Figure 3 shows a block diagram of the GS CIS and pixel circuit schematic. The chip comprises a photodiode array, column slope 12 bit ADCs with dual-gain amplifiers (SSDG-ADC) [8], column memories, signal processors and low-voltage differential signaling (LVDS) interface. The pixel array consists of 2676 (H) × 2200 (V) pixels. A unit pixel is configured as a two floating diffusion (FD) shared pixel structure with charge domain memories (MEM) and overflow gates (OG). GS is used for global charge transfer, TX is used for rolling signal readout, and OG is used to discharge carriers that exceed a photodiode full charge capacity.

## 3. Our Technology

**Figure 4:**
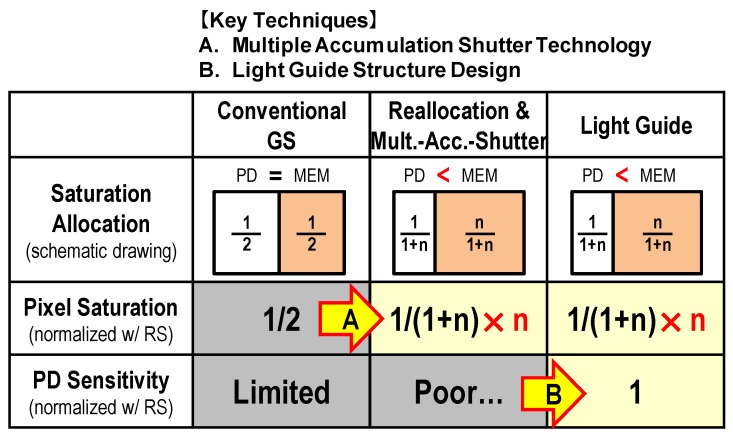
Key techniques.

Figure 4 shows the outline of our two key techniques to realize superior optical characteristics while suppressing the reduction of saturation more than the conventional GS pixels. The first technique is the multiple accumulation shutter technology. This technique improves pixel saturation. The second technique is the light guide structure. This technique improves optical performance. As a premise of adopting these two techniques, we first explain the idea of saturation allocation that is important in these techniques.

**Figure 5:**
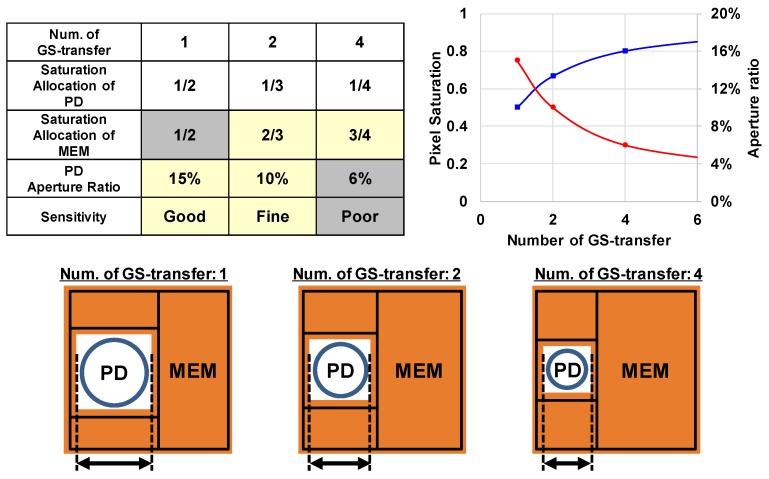
Allocation of saturation and the PD aperture ratio estimated from the layout according to the number of GS-transfer.

Figure 5 shows the allocation of saturation and the PD aperture ratio estimated from the layout according to the number of GS-transfer. The result of one-time GS-transfer indicates the maximum saturation decreases by about 25% compared to two-times GS-transfer, whereas the result of four-times GS-transfer indicates the saturation improves about 20% compared to two-times GS-transfer. However, the result of four-times GS-transfer indicates the aperture ratio will fall to below 10% and sensitivity will decrease. Particularly, the spectral sensitivity of red is remarkably reduced due to the diffraction limit. From this point of view, the developed GS pixel has adopted the saturation allocation of PD:MEM = 1:2. Additionally, as the MEM has a comparatively large area, so we can attain high well capacity without highly concentrated impurity doping. Thus, we have realized low-noise MEM configuration.

### 3.1. Multiple Accumulation Shutter Technology

**Figure 6:**
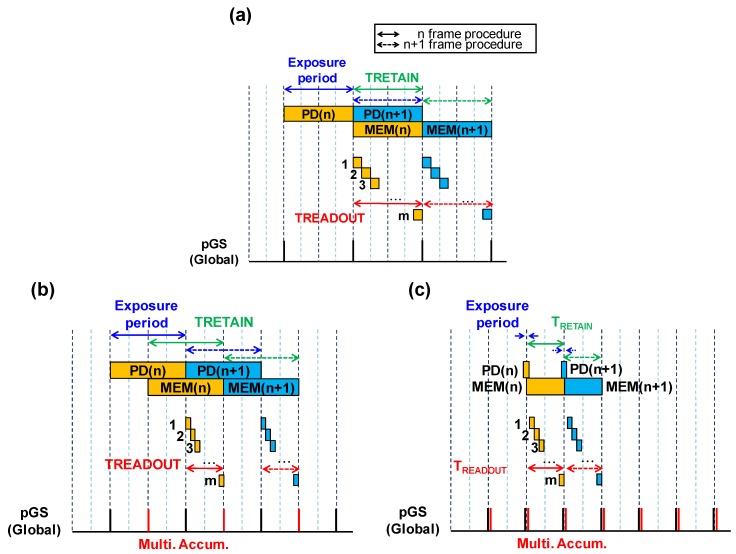
Signal readout procedure of (**a**) the conventional; (**b**) the multiple accumulation of seamless and (**c**) the multiple accumulation of non-seamless.

Figure 6a shows the conventional signal readout procedure of the GS CISs. During the signal readout period (T_READOUT_), signal charges generated in the photo conversion region corresponding to one frame exposure period are retained in the MEM. In this readout procedure, the time period for retaining the signal in the MEM (T_RETAIN_) is as long as the T_READOUT_. Besides, the signal transfer from PD to MEM is usually once in a frame. Consequently, the saturation signal of the GS pixel is determined by the PD saturation, which is as much as that of the MEM. Figure 6b shows the proposed multiple accumulation readout procedure [5,6]. One of the features of this procedure is that T_READOUT_ can be configured at a fraction of the T_RETAIN_. For example, the ratio (=T_READOUT_/T_RETAIN_) can be set as 1/2. As a consequence of this contrivance, signal readout from the MEM of each pixel is completed much sooner than conventional procedures. Once the signal readout from the MEM is completed, the MEM is ready to receive signal charges of the next frame. Thus, we can transfer PD signal to the MEM at an earlier time. After this signal transfer, the MEM still can retain signal charge until the next frame readout starts. As a result, the number of transfers from the PD to the MEM can be multiplied. In consequence, we can attain several times as much saturation signal as that of PD as a pixel saturation. In this readout procedure, light exposure and signal readout are performed simultaneously, hence the seamless signal accumulation can be carried out. Figure 6c shows the timing diagram of non-seamless signal accumulation with multiple accumulation readout procedure. One of the feature of this procedure is that the frame rate is almost equal to T_READOUT_ time. Therefore, this procedure is able to achieve higher frame rate than seamless signal accumulation procedure without decreasing saturation signal.

### 3.2. Light Guide Structure Design

**Figure 7:**
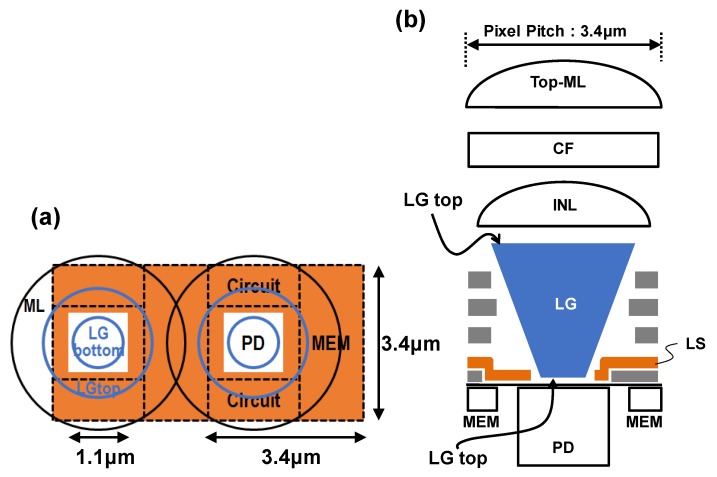
(**a**) Top-view and (**b**) the cross-section diagram of the pixel.

Figure 7a,b shows the top-view and the cross-section diagram of the pixel. The pixel pitch is 3.4 μm and the photodiode area is only about 25% of the pixel. Generally, in the GS pixel, there is a light shield (LS) structure to avoid parasitic light for the memory. The PD aperture size is limited by light shield placement. To guide the incident light for the photodiode, we adopted a high refractive index material based light guide structure. LG top diameter, LG bottom diameter and taper design greatly affect optical characteristics. In particular, the parasitic light sensitivity (PLS) must be taken into consideration. Additionally, the light guide structure has a problem that it becomes more difficult to manufacture the gentle slope structure. Therefore, this section is given a detailed description about the relation between light guide structure shape and optical characteristics.

**Figure 8:**
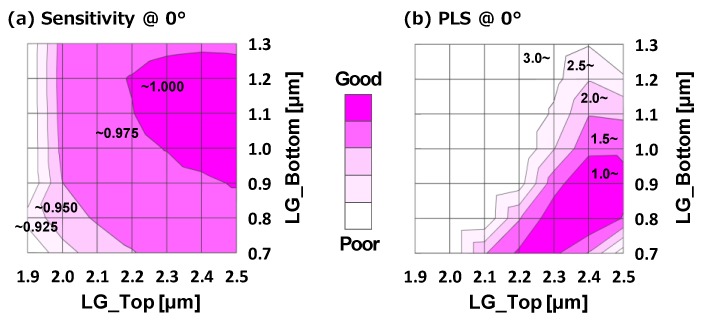
Simulated (**a**) sensitivity and (**b**) PLS results of light guide top and bottom diameter dependence.

Figure 8a,b shows simulation results of the sensitivity and the PLS in the case of the incident light angle at 0°. The simulation was carried out with single wavelength of 550 nm (green) based on the three-dimensional finite-difference time-domain (3D-FDTD) method. The horizontal axis is the top diameter and the vertical axis is the bottom diameter. These results indicate that the maximum value region is different between the sensitivity and the PLS. If we try to optimize the sensitivity, the LG top should be larger and the LG bottom should be around 1.0 µm. On the other hand, if we try to optimize the PLS, the LG top should be in the range of 2.3 µm to 2.5 µm and the LG bottom should be in the range of 0.8 µm to 0.9 µm and this range requires a gentle slope light guide structure. Therefore, we need to take account of these characteristics to design the light guide structure. 

**Figure 9:**
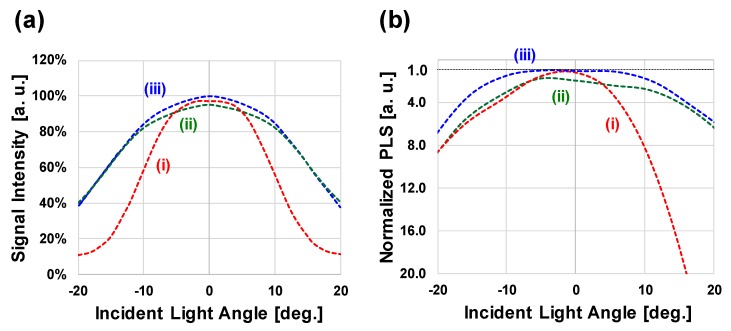
Simulated (**a**) sensitivity and (**b**) PLS results of incident light angle dependence.

Figure 9a,b shows the simulated results of incident light angle dependence of sensitivity and PLS, respectively. Simulation condition (i) is the result of the pixel without LG, Simulation condition (ii) is the result of the pixel with LG (Top: 2.0 µm, Bottom: 0.8 µm; steep slope), and Simulation condition (iii) is the result of the pixel with LG (Top: 2.4 µm, Bottom: 0.8 µm; gentle slope). The results of (i) show that both the sensitivity and PLS have very poor incident angle characteristics. On the other hand, in the case of result of (ii) and (iii), the incident angle characteristics are improved. However, in result of (ii), the characteristics of vertical incident light is inferior to that of (i). This is due to the influence of reflection and refraction that occurs when a part of the light not entering the aperture of the light guide structure enters from the light guide side face. Therefore, in order to realize high optical characteristics, a gentle slope light guide structure such as result (iii) is preferable. Table 1 summarizes these results.

## 4. Experimental Results

**Figure 10:**
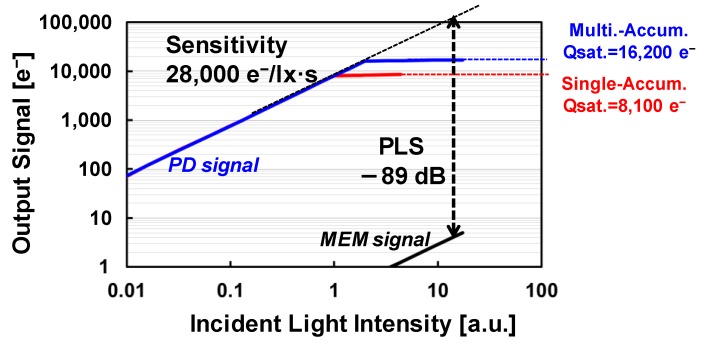
Measured output characteristics.

Figure 10 shows the measured photoelectric conversion characteristics of the GS pixel. The results shown in red are measured by single charge accumulation from the PD to the MEM. The saturation signal is 8100 e^−^. The results shown in green are measured by double charge accumulation from the PD to the MEM. In this experiment, the saturation signal of 16,200 e^−^ has been achieved. In addition, the sensitivity and the PLS are 28,000 e^−^/lx·s in green pixel and −89 dB, respectively. The measurement was implemented using CIE light source A (2856 K).

**Figure 11:**
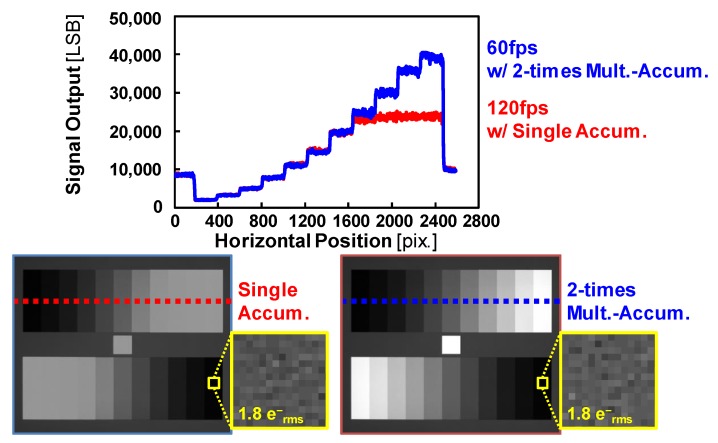
Measured output characteristics.

Figure 11 shows monochrome chart captures for dynamic range measurement and linearity measurement. Signal outputs versus horizontal position are plotted in the upper part of the figure for the two accumulation modes. In the bright area, the output of the single accumulation is saturated. The multiple-accumulation shutter technique is very effective for higher saturation. The noise level of the dark part is same in the both readout procedures. In our multiple accumulation, the noise level is not increased by using charge domain summation.

**Figure 12:**
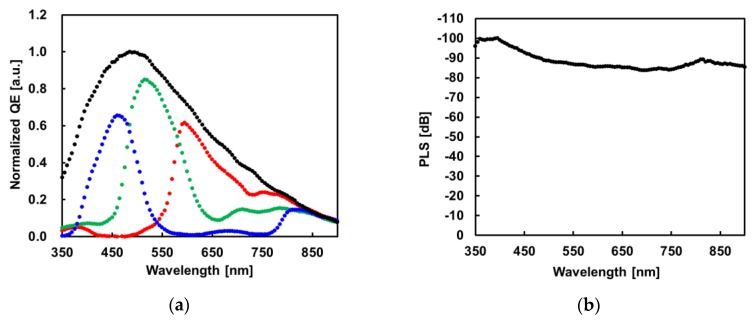
(**a**) Spectral sensitivity and (**b**) Spectral parasitic light sensitivity.

Figure 12a shows the quantum efficiency (QE) spectrum of red, green, blue and white pixels. The sensitivity of the red color region is sufficient in spite of the small photodiode aperture. Figure 12b shows the incident light wavelength dependence of PLS. Although the PLS generally drops off at the longer wavelength region, the developed GS pixel keeps higher PLS even in the NIR region. This result is important for automotive, industrial and other emerging applications.

**Figure 13:**
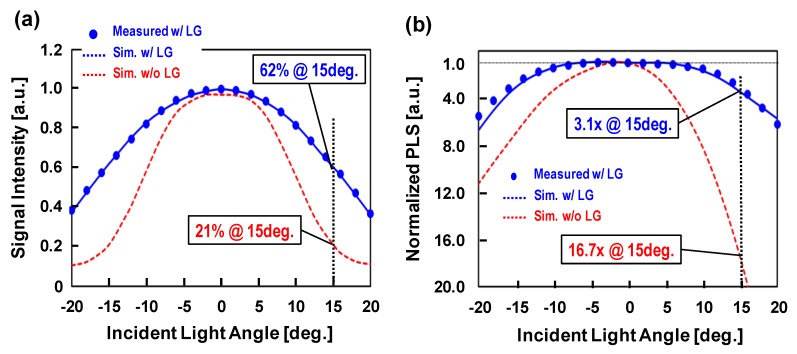
Measured and simulation results of the incident light angle dependence of (**a**) sensitivity and (**b**) PLS.

Figure 13a shows the measured and simulation results of the incident light angle dependence of sensitivity with and without light guide structure normalized to sensitivity of perpendicular light. These results indicate that the ratio of 15° incident light to the perpendicular light has been improved from 21% to 62% by the effect of the light guide structure. Figure 13b shows the measured and simulation results of the incident light angle dependence of PLS with and without light guide structure normalized to PLS of perpendicular light. These results indicate that the ratio of 15° incident light to the perpendicular light has been improved from 16.7× to 3.1× by the effect of the light guide structure.

## 5. Conclusions

**Figure 14:**
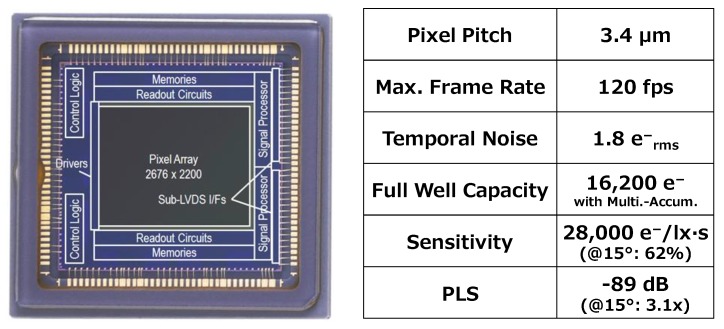
Chip microphotograph and pixel performance summary.

We have described our two key techniques, that is multiple accumulation shutter technology and gentle slope light guide structure. Furthermore, we have shown the importance of the light guide structure design in small GS pixel. The developed GS pixel attains 1.8 e^−^ temporal noise and 16,200 e^−^ full well capacity in 120 fps operation with multiple accumulation shutter technology. The sensitivity and PLS are 28,000 e^−^/lx·s and −89 dB. Moreover, the gentle slope light guide structure is effective for the incident light angle dependence of sensitivity and PLS. Figure 14 shows a chip microphotograph and pixel performance summary. The size of optical format is two to three inches.

Table 2 shows a performance comparison among recently published CISs. The measured chip power consumption is 450 mW at a frame rate of standard 120 fps mode and 60 fps with the multiple-accumulation shutter. In each mode, FOM1 of 1.27 e^−^·nJ and 2.54 e^−^·nJ are achieved, respectively, when FOM1 is defined as (power × noise × 109)/(number of effective pixels × fps). Furthermore, FOM2 of 0.28 e^−^·pJ and 0.29 e^−^·pJ are achieved, respectively, when FOM2 is defined as (power × noise × 1012)/(number of effective pixels × fps)/(full well capacity/noise). Based on recent results in Table 2, the FOMs of the fabricated image sensor are comparable or better than those of others, in spite of the addition of the GS function and the pixel size shrinkage.

## Figures and Tables

**Table 1:** Simulated optical performance summary.

		(i) Non-LG	(ii) Steep LG	(iii) Gentle LG
Top diameter		-	2.0	2.4
Bottom diameter		-	0.8	0.8
Taper		-	Steep	Gentle
Signal Intensity	@ 0°	98%	97%	100%
	@ 15°	21%	62%	62%
Normalized PLS	@ 0°	1.2×	2.7×	1.0×
	@ 15°	16.7×	4.0×	3.1×

**Table 2:** Summarized specifications and characteristics comparison.

	Unit	This work	[5] IEDM 2016			[9] VLSI 2016	[10] IISW 2013
Shutter Function	-	GS	GS			GS	GS
Pixel Pitch	μm	3.4	6.4			5.86	5.0
Number of Effective Pixels	-	2592 × 2054	4046 × 2496			3840 × 2164	1920 × 1080
Maximum Frame Rate	fps	120	30	60	90	480	240
Full Well Capacity	e^−^	1620	70,000	38,000	19,000	30,450	15,000
	e^−^/µm^2^	1380	1700	930	460	890	600
Sensitivity	e^−^/lx·s	28,000	80,000			17,500	54,250
	e^−^/lx·s/um2	2420	1950			510	2170
Temporal Noise	e^−^_rms_	1.8	1.8			4.6	4.0
Dynamic Range	dB	79.0	92.0	86.0	80.0	76.3	71.5
Parasitic Light Sensitivity	dB	−89	−78			−100	−70
Power Consumption	W	0.45	1.5			5.23	1.1
Figure of Merit 1	e^−^·nJ	1.27	8.91	4.46	2.23	6.04	8.84
Figure of Merit 2	e^−^·nJ	0.14	0.23	0.21	0.21	0.92	2.36

FoM1= Power[W] × DRN[e^−^] × 109/(FPS[s-1] × Num. of Eff. Pixels).

FoM2= Power[W] × DRN[e^−^] × 1012/(FPS[s-1] × Num. of Eff. Pixels × DRU); DRU=FWC[e^−^]/DRN[e^−^].
